# Impact of simple equation for estimating appendicular skeletal muscle mass in patients with stable coronary artery disease undergoing percutaneous coronary intervention^☆^

**DOI:** 10.1016/j.ijcha.2022.101163

**Published:** 2022-12-12

**Authors:** Ryota Nishio, Tomotaka Dohi, Tatsuya Fukase, Mitsuhiro Takeuchi, Norihito Takahashi, Hirohisa Endo, Shinichiro Doi, Iwao Okai, Hiroshi Iwata, Shinya Okazaki, Katsumi Miyauchi, Hiroyuki Daida, Tohru Minamino

**Affiliations:** a Department of Cardiovascular Biology and Medicine, Juntendo University Graduate School of Medicine, Tokyo, Japan; b Japan Agency for Medical Research and Development-Core Research for Evolutionary Medical Science and Technology (AMED-CREST), Japan Agency for Medical Research and Development, Tokyo, Japan

**Keywords:** Percutaneous coronary intervention, Coronary artery disease, Appendicular skeletal mass index, ASM, appendicular skeletal muscle mass, ASMI, appendicular skeletal muscle mass index, AWGS, Asian Working Group for Sarcopenia, BIA, bioelectrical impedance analysis, CAD, coronary artery disease, CKD, chronic kidney disease, CI, confidence interval, CVD, cardiovascular deaths, DXA, dual-energy X-ray absorptiometry, HR, hazard ratio, LVEF, left ventricular ejection fraction, MACE, major adverse cardiac events, PCI, percutaneous coronary intervention

## Abstract

**Background:**

Sarcopenia, which is evaluated based on appendicular skeletal muscle mass (ASM) using dual-energy X-ray absorptiometry and bioelectrical impedance analysis, is a prognostic predictor for adverse outcomes in patients with coronary artery disease (CAD). However, a simple equation for estimating ASM is yet to be validated in clinical practice.

**Methods:**

We enrolled 2211 patients with CAD who underwent percutaneous coronary intervention at our hospital between 2010 and 2017. The mean age was 68 years and 81.5 % were men. Patients were divided into 2 groups based on each ASM index (ASMI): low; male < 7.3 and female < 5.0 and high; male ≥ 7.3 and female ≥ 5.0. ASM was calculated using the following equation: 0.193 × bodyweight + 0.107 × height − 4.157 × gender − 0.037 × age − 2.631. Primary endpoints were major adverse cardiac events (MACE, which includes cardiovascular death, non-fatal myocardial infarction, non-fatal stroke, and hospitalization for heart failure), and all-cause mortality.

**Results:**

During the median follow-up period of 4.8 years, cumulative incidence of events were significantly higher in the low ASMI group. Cox proportional hazards model revealed that the low ASMI group had a significantly higher risk of primary endpoints than the high ASMI group (all-cause mortality; hazard ratio (HR): 2.13, 95 % confidence interval [CI]: 1.40–3.22, p < 0.001 and 4-point MACE; HR: 1.72, 95 % CI: 1.12–2.62, p = 0.01). Similar trends were observed after stratification by age of 65 years.

**Conclusion:**

Low ASMI, evaluated using the aforementioned equation, is an independent predictor of MACE and all-cause mortality in patients with CAD.

## 1 Introduction

Globally, coronary artery disease (CAD) is a leading cause of mortality and cardiovascular deaths (CVD) are increasing due to an aging population [1], [2], [3]. Aging is associated with changes in muscle quantity and quality. The European Working Group on Sarcopenia in Older People and the Asian Working Group for Sarcopenia (AWGS) recommends diagnosis of sarcopenia based on the presence of low muscle strength, low physical performance, and low appendicular skeletal muscle mass (ASM) [4], [5]. Sarcopenia has negative consequences, including motor and physical disabilities, reduced quality of life, and mortality [6], [7]. In addition, it is a prognostic factor in patients with heart failure and cardiovascular disease [8], [9], [10], [11] and is a factor that exacerbates the metabolic syndrome [12]. Skeletal muscle mass, a diagnostic parameter of sarcopenia, accounts for almost half of body weight and is vital in various metabolic pathways (i.e., insulin resistance, arterial stiffness, and oxidative stress) [13], [14], [15]. In Asia, dual-energy X-ray absorptiometry (DXA) and bioelectrical impedance analysis (BIA) are frequently used to measure skeletal muscle mass. The AWGS has established cutoff values for DXA and BIA and recommended the use of either modality to measure muscle mass to diagnose sarcopenia [4]. Recently, equations for estimating ASM using height, weight, sex, and age have been proposed for the Asian population. The estimation equation was developed based on skeletal muscle mass measured via DXA in Chinese adults aged 18– 69 years [16], [17]. However, whether ASM calculated from the estimation equation is a prognostic factor in patients with CAD has not yet been assessed. This study investigated whether the ASM equation can predict the prognosis of patients with CAD undergoing percutaneous coronary intervention (PCI).

## 2 Methods

In this single-center observational retrospective cohort study, 2,211 consecutive patients with CAD who underwent PCI (first-time) from 2010 to 2017 were enrolled. The data of patients with measurements of the appendicular skeletal muscle mass (ASM) and ASM index (ASMI) on day of admission for PCI were analyzed. The ASM was estimated using an equation previously validated for the Asian population [4], [17].

ASM = 0.193 × body weight + 0.107 × height − 4.157 × gender − 0.037 × age − 2.631

(Weight in kg; height in m; age in years; gender: 1, for men and 2, for women).

ASMI = ASM / height^2^ (height in m).

**Fig. 1:**
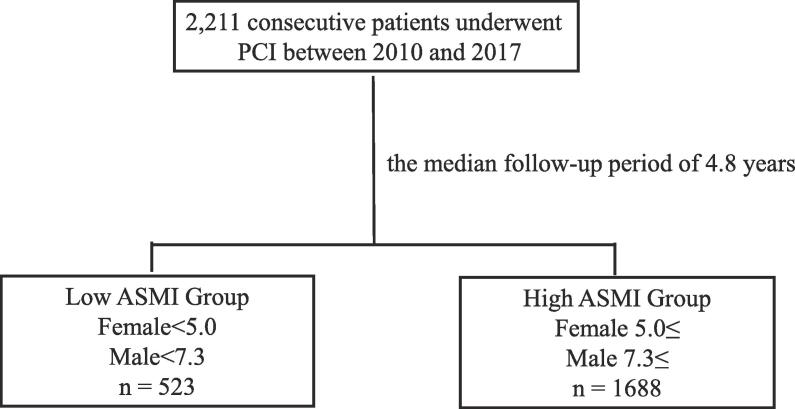
Study flow chart. A total of 2211 consecutive patients with stable CAD who underwent PCI (first) from 2010 to 2017 at the Juntendo University Hospital. All patients were divided into 2 groups based on the ASMI. CAD, coronary artery disease; PCI, percutaneous coronary intervention; ASMI, appendicular skeletal muscle mass index.

Low ASMI was defined as the lowest 20th percentile of the study population [16]. The cut-off values were < 7.3 kg/m^2^ and < 5.0 kg/m^2^ in men and women, respectively. Patients were divided into 2 groups based on the ASMI: low ASMI; male < 7.3 and female < 5.0 and high ASMI; male ≥ 7.3 and female ≥ 5.0 (Fig. 1). Demographic data and information on coronary risk factors, medications, revascularization procedure-related factors, and comorbidities were collected to create a database.

Blood samples were collected early morning on the day of PCI after overnight fasting. Blood pressure (BP) was measured on admission. Patients with BP of > 140/90 mmHg and/or those on antihypertensive medications were considered hypertensive (HT). Dyslipidemia (DL) was defined based on the values of low-density lipoprotein cholesterol (LDL-C), high-density lipoprotein cholesterol (HDL-C), and triglycerides (TG), (≥140, ≤40, and ≥ 150 mg/dL, respectively) or undergoing treatment with statins and/or lipid-lowering agents [18]. Diabetes mellitus (DM) was defined as either hemoglobin A1c (HbA1c) level of ≥ 6.5 % or medication with insulin or oral hypoglycemic drugs. Chronic kidney disease (CKD) was defined as an estimated glomerular filtration rate of < 60 mL/min/1.73 m^2^, calculated using the Modification of Diet in Renal Disease equation modified with a Japanese coefficient using the follow-up serum creatinine level [19]. A “current smoker” was defined as a person who was a smoker at the time of PCI or had quit smoking ≤ 1 year before PCI [20].

The primary endpoints of this study were major adverse cardiac events (MACE) and all-cause mortality. MACE was defined as a composite of CVD, non-fatal myocardial infarction (MI), non-fatal stroke, and hospitalization for heart failure. CVD was defined as death caused by MI or heart failure, or sudden death. Survival data and information about MACE was obtained by contacting the patients and accessed from their medical records. Mortality data were collected from the patients’ families, and details of events associated with the cause of death, from hospitals where the patient had been admitted. All data were collected by blinded investigators. Time to event was measured from the date of the first PCI.

Categorical data were presented as numbers and percentages and compared using the chi-square test. Continuous variables were expressed as mean ± standard deviation or as median and interquartile range and compared using one-way analysis of variance (Kruskal–Wallis test), which was applied to the 4 groups. Unadjusted cumulative event rates were estimated using Kaplan–Meier curves and compared between the 2 groups. Additionally, patients were stratified by age using 65 years as the cutoff level in the Kaplan–Meier analysis. Associations between ASMI and the primary endpoint were determined using multivariate Cox proportional hazard regression analysis. Model 1 was adjusted for the variables in HT, DL, DM, and CKD; model 2 was adjusted for the variables in model 1 plus left ventricular ejection fraction (LVEF), and multivessel disease. Furthermore, patients were stratified by age using 65 years as the cutoff level in the Cox proportional hazard regression analysis. Hazard ratios (HRs) and 95 % confidence intervals (CIs) were calculated; p-values < 0.05 were considered statistically significant. All statistical analyses were performed using JMP version 14.0 (SAS Institute, Cary, NC, USA).

## 3 Results

All patients who underwent the first PCI from 2010 to 2017 were enrolled and classified into 2 groups: low ASMI included 523 patients (23.7 %) and ASMI, 1688 patients (76.3 %). Table 1 presents the clinical characteristics of the patients. The mean ASMI values were 7.0 (6.8– 7.2) and 4.6 (4.4– 4.9) in males and females, respectively, in the low ASMI group and 8.0 (7.7–8.4) and 5.7 (5.4–6.2) in males and females, respectively, in the high ASMI group. The mean age of the patients was 67.5 ± 11.0 years. No significant differences were noted in gender, hypertension, diabetes, and acute coronary syndrome between the 2 groups. Low ASMI groups had a higher prevalence of CKD, multivessel disease, and higher value of brain natriuretic peptide (p < 0.01 for all). The high ASMI group exhibited a higher prevalence of dyslipidemia, family history of CAD, and higher value of LVEF, albumin, and HbA1c (p < 0.01 for all).

**Table 1:** Baseline clinical characteristics of patients.

	**Overall**	**Low ASMI**	**High ASMI**	
	**(n = 2211)**	**(n = 523)**	**(n = 1688)**	***p***
**Baseline characteristic**				
ASMI, kg/m2 (Male)	7.8 (7.3 – 8.2)	7.0 (6.8 – 7.2)	8.0 (7.7 – 8.4)	<0.001
ASMI, kg/m2 (Female)	5.6 (5.1 – 6.0)	4.6 (4.4–4.9)	5.7 (5.4 – 6.2)	<0.001
Age, years	67.5 ± 11.0	74.0 ± 8.8	65.5 ± 10.9	<0.001
Male, n (%)	1802 (81.5)	436 (83.4)	1366 (80.9)	0.2
BMI, kg/m2	24.3 ± 3.6	20.2 ± 1.7	25.5 ± 3.1	<0.001
Hypertension, n (%)	1611 (72.9)	368 (70.4)	1243 (73.6)	0.14
Dyslipidemia, n (%)	1662 (75.2)	321 (61.4)	1341 (79.4)	<0.001
Diabetes, n (%)	914 (41.3)	218 (41.7)	696 (41.2)	0.86
Current smoking, n (%)	509 (23.1)	105 (20.2)	404 (24.0)	0.07
CKD, n (%)	596 (30.0)	195 (37.3)	401 (23.8)	<0.001
Family history of CAD, n (%)	620 (28.0)	124 (23.8)	496 (29.6)	0.009
ACS, n (%)	639 (28.9)	146 (27.9)	493 (29.2)	0.57
LVEF, %	60.7 ± 12.2	58.4 ± 13.7	61.3 ± 11.6	<0.001
Multivessel disease, n (%)	1276 (57.7)	337 (65.8)	939 (56.4)	<0.001
**Medication**				
Aspirin, n (%)	2058 (93.4)	480 (92.0)	1578 (93.8)	0.14
β-blocker, n (%)	962 (44.1)	211 (40.8)	751 (45.1)	0.09
CCB, n (%)	875 (40.1)	209 (40.4)	666 (40.0)	0.86
ACE-I/ARB, n (%)	1038 (47.6)	229 (44.3)	809 (48.6)	0.09
Statin, n (%)	1792 (81.4)	388 (74.5)	1404 (83.6)	<0.001
**Baseline data**				
HbA1c, %	6.3 (6.2 – 6.3)	6.2 (6.1 – 6.2)	6.3 (6,2 – 6.3)	0.008
TG, mg/dL	134 (129–138)	99 (95–103)	144 (138–150)	<0.001
HDL-C, mg/dL	45 (44–45)	48 (47–49)	44 (43–44)	<0.001
LDL-C, mg/dL	100 (98–101)	97 (94–99)	101 (99–102)	0.02
BNP, pg/mL	141 (126–155)	243 (215–272)	107 (91–124)	<0.001
Alb, g/dL	3.8 (3.8 – 3.9)	3.7 (3.6 – 3.8)	3.9 (3.8 – 3.9)	<0.001
eGFR, mL/min/1.73 m2	71 (70–72)	64 (62–66)	74 (72–75)	<0.001

ACE-I, angiotensin-converting enzyme inhibitors; ACS, acute coronary syndrome; Alb, albumin; ARB, angiotensin receptor blockers; ASMI, appendicular skeletal muscle index; BMI, body mass index; BNP, B-type natriuretic peptide; CAD, coronary artery disease; CKD, chronic kidney disease; eGFR, estimated glomerular filtration rate; HbA1c, hemoglobin A1c; HDL-C, high-density lipoprotein cholesterol; LDL-C, low-density lipoprotein cholesterol; LVEF, left ventricular ejection fraction; RIR, residual inflammatory risk; TG, triglycerides.

**Fig. 2:**
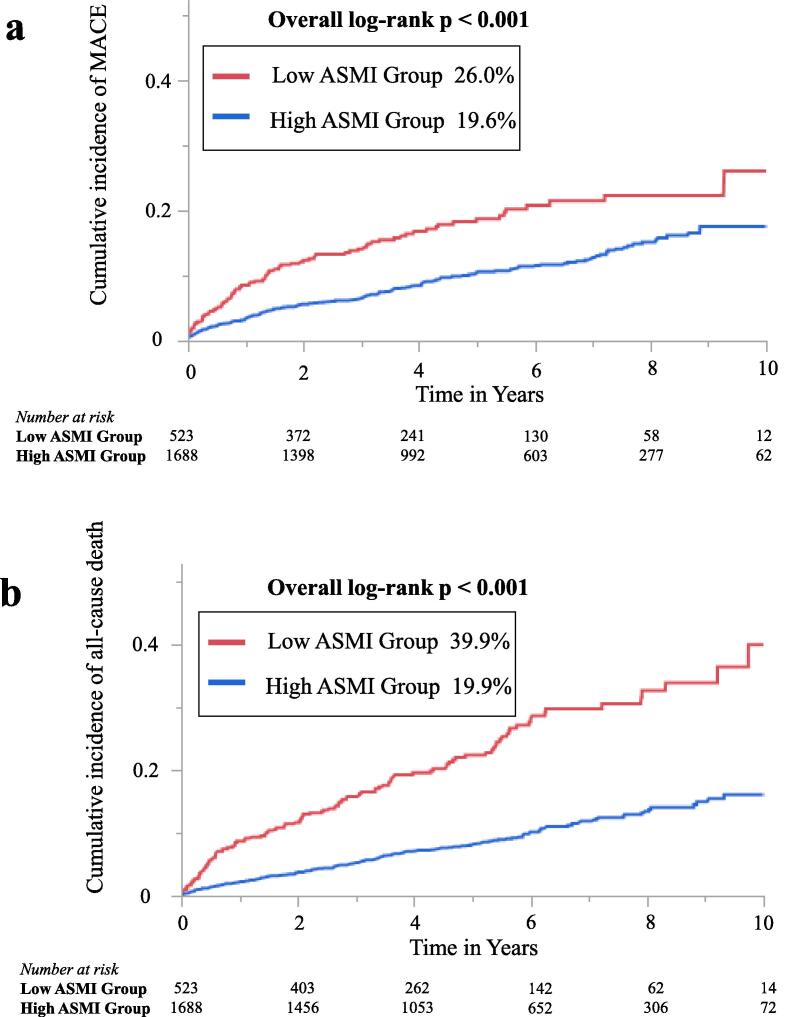
Kaplan–Meier curve for MACE (a) and all-cause mortality (b) in patients classified by ASMI. Kaplan–Meier curves demonstrate significant differences in all-cause mortality between the groups (both log-rank test, p < 0.001). ASMI, appendicular skeletal muscle mass index; MACE, major adverse cardiac events.

**Fig. 3:**
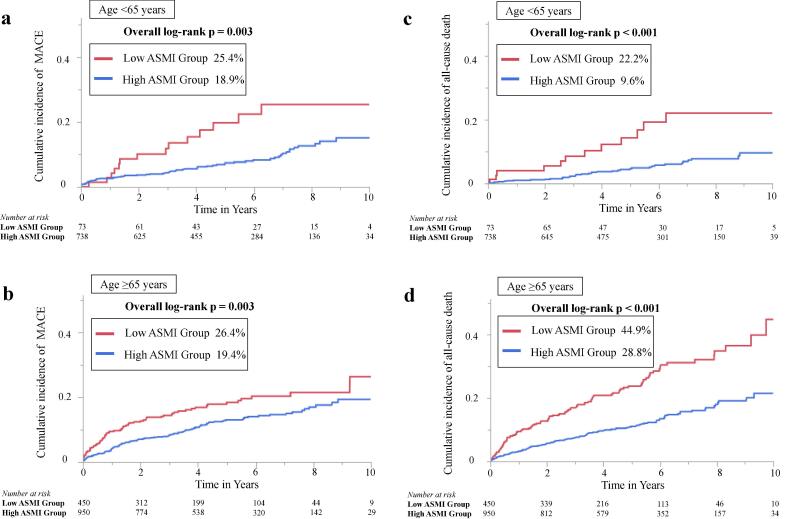
Kaplan–Meier curve for MACE among patients aged < 65 years (a); aged ≥ 65 years (b) and Kaplan–Meier curve for all-cause mortality among patients aged < 65 years (c); aged ≥ 65 years (d). Kaplan–Meier curves for MACE demonstrate significant differences in event rates between the groups, even when stratified by the age of 65 years (log-rank test, all p = 0.003). Kaplan–Meier curves for all-cause mortality demonstrate significant differences in event rates between the groups, even when stratified by the age of 65 years (log-rank test, all p < 0.001). ASMI, appendicular skeletal muscle mass index; MACE, major adverse cardiac events.

The association between ASMI and clinical parameters were examined. ASMI had a significant correlation with age (r = 0.46, p < 0.001) and BMI (r = 0.73, p < 0.001). Correlation between ASMI and albumin (r = 0.23, p < 0.001) was statistically significant, although relatively weak. The median follow-up period was 4.8 (interquartile range, 2.9–7.1) years. The median follow-up period for the low ASMI group was 4 (interquartile range, 2.1–6.2) years and for the high ASMI group was 5 (interquartile range, 3.1–7.3) years. In total, 266 (12.0 %) cases of MACE and 279 (12.6 %) cases of all-cause mortality were identified during the follow-up, including 77 (3.4 %), 49 (2.2 %), 60 (2.7 %), and 80 (3.6 %) cases of CVD, non-fatal MI, non-fatal stroke, and hospitalization for heart failure, respectively. In the low ASMI group, 88 (16.8 %) cases of MACE and 121 (23.1 %) cases of all-cause mortality were identified, and in the high ASMI group, 178 (10.5 %) cases of MACE and 158 (9.3 %) cases of all-cause mortality were observed. Fig. 2**a and 2b** illustrate the Kaplan–Meier curves for MACE and all-cause mortality. The cumulative incidences of MACE were significantly higher in the low ASMI group (26.0 % versus 19.6 %; log-rank p < 0.001). Furthermore, Kaplan–Meier curves for MACE revealed significant differences in the incidence of events between the 2 groups, even when stratified by the age of 65 years (<65 age group: log-rank p = 0.003; ≥65 age group: log-rank p = 0.003) (Fig. 3**a and 3b**). In addition, the cumulative incidences of all-cause mortality were significant higher in the low ASMI group (39.9 % versus 19.9 %; log-rank p < 0.001). Despite stratification by the age of 65 years, Kaplan–Meier curves for all-cause mortality revealed significant differences in the incidence of events between the 2 groups (<65 age group: log-rank p < 0.001; ≥65 age group: log-rank p < 0.001) (Fig. 3**c and 3d**). Supplemental Table 1 shows the baseline clinical characteristics of patients stratified into two age groups (65 years was set as the cut-off age). There were no significant differences in LVEF, multivessel disease, statin use, and albumin between low ASMI group and high ASMI group in < 65 years age group. In ≥ 65 years age group, high ASMI group had significantly higher rates of HT and higher rates of angiotensin-converting enzyme inhibitors and angiotensin receptor blockers medications than low ASMI group. The Kaplan-Meier curve was stratified by age tertiles: Tertile 1: <64 years age group, Tertile 2: 64–73 years age group, and Tertile 3: ≥74 years age group. MACE and all-cause mortality were significantly higher in the low ASMI group for Tertile 1 and Tertile 3. In Tertile 2, all-cause mortality was higher in the low ASMI group, whereas, there was no significant difference in MACE.

Table 2 summarizes the results of the Cox proportional hazard regression analysis for MACE and all-cause mortality. The low ASMI group was significantly associated with MACE than the high ASMI group (HR 1.65; 95 % CI 1.20–2.24; p = 0.01), even after adjustment for other risk factors (HT, DL, DM, CKD, LVEF, and multivessel disease). Furthermore, the risk of all-cause mortality was significantly higher in patients in the low ASMI group after adjustment for vital covariates (HR 2.79; 95 % CI 2.09–3.70; p < 0.001). Even after adjusting the significant differences in patient backgrounds between the two groups, the low ASMI group was significantly associated with MACE (HR 1.76; 95 % CI 1.15–2.68; p = 0.009) and all-cause death (HR 2.20; 95 % CI 1.45–3.33; p = 0.002). In Cox regression analysis, there was no significant interaction between ASMI and gender (MACE: p for interaction = 0.51, all-cause death: p for interaction = 0.31). Table 3 presents the results of the Cox proportional hazard regression analysis for primary endpoints stratified by the age of 65 years. In the < 65 years age group, multivariate Cox hazard analysis demonstrated that the low ASMI group had a significantly higher risk of MACE than the high ASMI group (HR 2.93; 95 % CI 1.41–5.59; p = 0.005). In the ≥ 65 years patients, the low ASMI group had a higher risk of MACE (HR 1.38; 95 % CI 0.96–1.97; p = 0.08). Furthermore, in both age groups, all-cause mortality was significantly higher in the low ASMI group, even after adjusting for other risk factors (>65 age group: HR 3.61; 95 % CI 1.58–7.51; p < 0.001 and > 65 age group: HR 2.24; 95 % CI 1.64–3.06; p < 0.001).

**Table 2:** Cox proportional hazard model for MACE and all-cause death.

Event	MACE		all-cause death	
	HR (95 % CI)	p	HR (95 % CI)	p
(Ref; High ASM index)				
Crude	1.96 (1.29–2.98)	0.002	2.90 (2.29–3.68)	< 0.001
Model 1	1.58 (1.21–2.05)	< 0.001	2.53 (1.98–3.23)	< 0.001
Model 2	1.65 (1.20–2.24)	0.01	2.79 (2.09–3.70)	< 0.001
Model 3	1.76 (1.15–2.68)	0.009	2.20 (1.45–3.33)	0.002

Model 1: adjusted for HT, DL, DM, CKD.

Model 2: adjusted for model 2 + LVEF, MVD.

Model 3: adjusted for age, gender, BMI, DL, CKD, LVEF, MVD.

HR, Hazard ratio; CI confidence interval, BMI, body mass index; CKD, chronic kidney disease; DM, diabetes mellitus; DL, dyslipidemia; HT, hypertension; LVEF, left ventricular ejection fraction; MACE, major adverse cardiac event; MVD, multi vessel disease.

**Table 3:** Cox proportional hazard model for MACE and all-cause death stratified age of 65 years.

**Event**	**MACE**		**all-cause death**	
	**HR (95** % **CI)**	**p**	**HR (95** % **CI)**	**p**
Age < 65 years (Ref; High ASM index)				
Crude	2.33 (1.25–4.03)	0.009	3.20 (1.60–5.95)	0.002
Model 1	2.14 (1.14–3.75)	0.02	2.82 (1.40–5.32)	0.005
Model 2	2.93 (1.41–5.59)	0.005	3.61 (1.58–7.51)	< 0.001
Age ≥ 65 years (Ref; High ASM index)				
Crude	1.55 (1.15–2.07)	0.004	2.28 (1.75–2.96)	< 0.001
Model 1	1.42 (1.05–1.91)	0.02	2.13 (1.63–2.78)	< 0.001
Model 2	1.38 (0.96–1.97)	0.08	2.24 (1.64–3.06)	< 0.001

Model 1: adjusted for HT, DL, DM, CKD.

Model 2: adjusted for model 1 + LVEF, MVD.

HR, Hazard ratio; CI confidence interval, CKD, chronic kidney disease; DM, diabetes mellitus; DL, dyslipidemia; HT, hypertension; LVEF, left ventricular ejection fraction; MACE, major adverse cardiac event; MVD, multi vessel disease.

## 4 Discussion

We investigated the relationship between long-term prognosis (median 4.8 years) and estimated ASMI in patients undergoing PCI for CAD. The major findings of the present study are as follows: (1) the rates of MACE in the low ASMI group were significantly higher than those in high ASMI group. Even after adjusting for vital covariates, low ASMI was associated with a higher incidence of MACE; (2) all-cause mortality was higher in the low ASMI group, and low ASMI was an independent predictor of all-cause mortality; (3) in the < 65 years group, low ASMI was a significant predictor of MACE and all-cause mortality. In addition, the HR for MACE in ≥ 65 years group was lower than that in the < 65 years group.

Low skeletal muscle mass has been reported as a potential prognostic predictor for CAD because of its association with decreased cardiopulmonary function, reduced exercise capacity, and arteriosclerosis [21], [22], [23], [24]. These studies have used CT and DXA to measure skeletal muscle mass. Although these methods provide a more accurate measurement, additional radiation exposure to patients who have undergone coronary angiography or PCI may be a critical issue. Therefore, measurement of skeletal muscle mass is not routinely performed in real-world clinical practice. In this study, we examined the prognosis of CAD patients using a simple equation for estimating ASMI. This estimated ASMI is a potential prognostic predictor of MACE and all-cause mortality in patients with CAD; it can help identify high-risk populations and may contribute to improved clinical outcomes.

Exercise therapy for sarcopenia has been reported to improve skeletal muscle mass and strength and gait speed [25] Several guidelines recommend cardiovascular rehabilitation especially for patients with CAD undergoing PCI [26], [27]. Increased daily activity in patients with CAD, including acute coronary syndrome, was closely associated with lower all-cause and cardiovascular mortalities, and has been reported to improve the quality of life and reduce mortality [28], [29]. However, the effect of cardiac rehabilitation on improving prognosis in patients with stable angina is unclear [30], [31]. Our study included approximately 70 % of patients with stable angina. This was considered necessary because these patients, particularly those with low ASMI, would benefit more from cardiac rehabilitation. Although in this study, we were only able to assess low ASMI, as one of the indicators of sarcopenia, our results indicate that low ASMI may be a potential therapeutic target to reduce adverse clinical outcomes in patients with CAD. In addition, cardiac rehabilitation has been reported to be safe when performed early after PCI [32], [33]. Thus, early exercise initiation as a non-pharmacologic therapy may prevent further progression of sarcopenia and lead to better clinical outcomes in patients with CAD. In our study, accurately determining the changes in skeletal muscle mass over time was challenging due to the nature of the estimation equation. Hence, further studies are needed to determine the prognostic value of improved skeletal muscle mass.

Sarcopenia has been reported as a poor prognostic factor in elderly patients [6], [34], [35]. Our study indicates that low ASMI may be a poor prognostic factor even in patients < 65 years. However, HR for MACE in ≥ 65 years group was lower than that in the < 65 years group. These results suggest that not only low skeletal muscle mass but also other indicators of sarcopenia, such as low muscle strength and low physical activity level, may play a role in prognosis in ≥ 65 years patients. In our study, we did not measure 6-minute walk or grip strength, and these factors need to be evaluated and analyzed additionally in the future, in order to evaluate the improvement of the prognosis for ≥ 65 years patients with CAD. Exercise therapy for elderly patients with CAD was reported to improve exercise tolerance and coronary risk factors as much as that of non-elderly patients [36], [37]. In addition to exercise therapy, there were also reports that suggested supplementation with essential amino acids and protein as nutritional therapy improves skeletal muscle mass [25]. In ≥ 65 years patients, past exercise habits (20 to 50 years old), usual number of steps, and physical activity level of 3 metabolic equivalents of task (METs) were associated with skeletal muscle mass and the development of sarcopenia. Exercise, nutrition, and lifestyle interventions may be effective in improving outcomes for ≥ 65 years patients with low ASMI.

Patients under 65 years of age were more strongly affected by low ASMI than patients over 65 years of age were affected. Regarding the association between sarcopenia and lifestyle-related diseases, skeletal muscle mass is reported to be lower in patients with type 2 diabetes [38], [39]. A report from the United States indicates that sarcopenia is related to glucose metabolism independent of obesity and this tendency is stronger in those aged < 60 years; moreover, reduced skeletal muscle mass may be a predictor of diabetes mellitus [40]. In addition to glucose metabolism, a relationship between sarcopenia and metabolic syndrome has been reported [41], [42], possibly because skeletal muscles secrete myokines, which increase insulin sensitivity, affect muscle physiology, and regulate the metabolism [43], [44], [45]. Nutritional therapy is an important part of treatment in patients with diabetes. Japanese guidelines do not set energy-producing nutrient ratios (carbohydrate, protein, and fat) for prevention and management of diabetes. Total energy intake is determined based on each patient's weight and BMI for weight loss [46]. However, it was reported that low energy intake in patients with diabetes decreases skeletal muscle mass [47]. Excessive energy restriction may induce sarcopenia in patients with low ASMI. In our study, low ASMI in patients aged < 65 years was a poor prognostic factor in multivariate analysis adjusting for cardiovascular risk at the time of PCI. In addition to perioperative complications, correction of metabolic syndrome and follow-up of abnormal metabolism was considered important after PCI in patients with low ASMI. Therefore, introducing nutritional and exercise guidance to increase skeletal muscle mass may be effective even in younger patients.

This study has a few limitations. First, the ASM estimation equation used in this study was not specific to the Japanese population and there is no consensus in the interpretation of skeletal muscle assessment. Hence, further knowledge is needed, and this can easily be obtained without additional radiation burden. Second, sarcopenia is defined based on the presence of low muscle strength, low physical performance, and low ASM. However, low muscle strength and low physical performance could not be evaluated in this study. In the future, it is necessary to examine the significance of estimated skeletal muscle mass considering the diagnostic criteria for sarcopenia. Third, unknown confounders might have affected outcomes regardless of analytical adjustments because this was a single-center observational study of a small-sized patient cohort. Therefore, further multicenter studies with a larger population should be conducted to provide greater statistical power and confirm the reproducibility of the results.

## 5 Conclusion

Low ASMI evaluated by a simple equation is an independent predictor of MACE and all-cause mortality in patients with CAD. Exercise therapy, nutritional therapy, and lifestyle modifications are considered important for patients with low ASMI.

## Trial registration

UMIN Unique trial Number: UMIN 000035587.

## Declarations

**Funding:** Not applicable.

**Ethics approval:** The ethics committee of Juntendo Clinical Research and Trial Center approved this study (IRB number 17-0206).

**Consent to participate and for publication:** Written informed consent to participate was obtained from all patients.

**Availability of data and material:** The datasets during and/or analyzed during the current study are available from the corresponding author with reasonable request.

**Code availability:** The relevant SAS codes for the statistical analysis are available from the corresponding author with reasonable request.

**Financial support:** This research received no external funding.

## Declaration of Competing Interest

The authors declare that they have no known competing financial interests or personal relationships that could have appeared to influence the work reported in this paper.

## References

[1] Hartley A., Marshall D.C., Salciccioli J.D., Sikkel M.B., Maruthappu M., Shalhoub J. (2016). Trends in Mortality From Ischemic Heart Disease and Cerebrovascular Disease in Europe: 1980 to 2009. Circulation.

[2] Lozano R., Naghavi M., Foreman K., Lim S., Shibuya K., Aboyans V. (2012). Global and regional mortality from 235 causes of death for 20 age groups in 1990 and 2010: a systematic analysis for the Global Burden of Disease Study 2010. Lancet.

[3] Roth G.A., Forouzanfar M.H., Moran A.E., Barber R., Nguyen G., Feigin V.L. (2015). Demographic and epidemiologic drivers of global cardiovascular mortality. N. Engl. J. Med..

[4] L.K. Chen, J. Woo, P. Assantachai, T.W. Auyeung, M.Y. Chou, K. Iijima, et al., Asian Working Group for Sarcopenia: 2019 Consensus Update on Sarcopenia Diagnosis and Treatment, J. Am. Med. Dir. Assoc. 21(3) (2020) 300-7.e2. 10.1016/j.jamda.2019.12.012 32033882

[5] Cruz-Jentoft A.J., Baeyens J.P., Bauer J.M., Boirie Y., Cederholm T., Landi F. (2010). Sarcopenia: European consensus on definition and diagnosis: Report of the European Working Group on Sarcopenia in Older People. Age Ageing.

[6] Yuki A., Ando F., Otsuka R., Shimokata H. (2017). Sarcopenia based on the Asian Working Group for Sarcopenia criteria and all-cause mortality risk in older Japanese adults. Geriatr. Gerontol. Int..

[7] Cruz-Jentoft A.J., Landi F., Topinková E., Michel J.P. (2010). Understanding sarcopenia as a geriatric syndrome. Curr. Opin. Clin. Nutr. Metab. Care..

[8] Springer J., Springer J.I., Anker S.D. (2017). Muscle wasting and sarcopenia in heart failure and beyond: update 2017. ESC Heart Failure..

[9] Rossignol P., Masson S., Barlera S., Girerd N., Castelnovo A., Zannad F. (2015). Loss in body weight is an independent prognostic factor for mortality in chronic heart failure: insights from the GISSI-HF and Val-HeFT trials. Eur. J. Heart Fail..

[10] Lavie C.J., De Schutter A., Patel D., Artham S.M., Milani R.V. (2011). Body composition and coronary heart disease mortality–an obesity or a lean paradox?. Mayo Clin. Proc..

[11] Kouvari M., Chrysohoou C., Dilaveris P., Georgiopoulos G., Magkas N., Aggelopoulos P. (2019). Skeletal muscle mass in acute coronary syndrome prognosis: Gender-based analysis from Hellenic Heart Failure cohort. Nutr. Metab. Cardiovasc. Dis..

[12] Hunter G.R., Singh H., Carter S.J., Bryan D.R., Fisher G. (2019). Sarcopenia and Its Implications for Metabolic Health. J. Obes..

[13] Im I.J., Choi H.J., Jeong S.M., Kim H.J., Son J.S., Oh H.J. (2017). The association between muscle mass deficits and arterial stiffness in middle-aged men. Nutr. Metab. Cardiovasc. Dis..

[14] Srikanthan P., Horwich T.B., Tseng C.H. (2016). Relation of Muscle Mass and Fat Mass to Cardiovascular Disease Mortality. Am. J. Cardiol..

[15] Kim Y., Han B.D., Han K., Shin K.E., Lee H., Kim T.R. (2015). Optimal cutoffs for low skeletal muscle mass related to cardiovascular risk in adults: The Korea National Health and Nutrition Examination Survey 2009–2010. Endocrine.

[16] Yang M., Hu X., Wang H., Zhang L., Hao Q., Dong B. (2017). Sarcopenia predicts readmission and mortality in elderly patients in acute care wards: a prospective study. J. Cachexia. Sarcopenia Muscle.

[17] Wen X., Wang M., Jiang C.M., Zhang Y.M. (2011). Anthropometric equation for estimation of appendicular skeletal muscle mass in Chinese adults. Asia Pac. J. Clin. Nutr..

[18] Teramoto T., Sasaki J., Ishibashi S., Birou S., Daida H., Dohi S. (2013). Diagnostic criteria for dyslipidemia. J. Atheroscler. Thromb..

[19] Matsuo S., Imai E., Horio M., Yasuda Y., Tomita K., Nitta K. (2009). Revised equations for estimated GFR from serum creatinine in Japan. Am. J. Kidney Dis..

[20] Miyauchi K., Morino Y., Tsukahara K., Origasa H., Daida H. (2010). The PACIFIC (Prevention of AtherothrombotiC Incidents Following Ischemic Coronary attack) Registry: Rationale and design of a 2-year study in patients initially hospitalised with acute coronary syndrome in Japan. Cardiovasc. Drugs Ther..

[21] Nichols S., O'Doherty A.F., Taylor C., Clark A.L., Carroll S., Ingle L. (2019). Low skeletal muscle mass is associated with low aerobic capacity and increased mortality risk in patients with coronary heart disease - a CARE CR study. Clin. Physiol. Funct. Imaging..

[22] Matsubara Y., Matsumoto T., Inoue K., Matsuda D., Yoshiga R., Yoshiya K. (2017). Sarcopenia is a risk factor for cardiovascular events experienced by patients with critical limb ischemia. J. Vasc. Surg..

[23] Ko B.J., Chang Y., Jung H.S., Yun K.E., Kim C.W., Park H.S. (2016). Relationship Between Low Relative Muscle Mass and Coronary Artery Calcification in Healthy Adults. Arterioscler. Thromb. Vasc. Biol..

[24] Atkins J.L., Whincup P.H., Morris R.W., Lennon L.T., Papacosta O., Wannamethee S.G. (2014). Sarcopenic obesity and risk of cardiovascular disease and mortality: a population-based cohort study of older men. J. Am. Geriatr. Soc..

[25] Yoshimura Y., Wakabayashi H., Yamada M., Kim H., Harada A., Arai H. (2017). Interventions for Treating Sarcopenia: A Systematic Review and Meta-Analysis of Randomized Controlled Studies. J. Am. Med. Dir. Assoc..

[26] Piepoli M.F., Hoes A.W., Agewall S., Albus C., Brotons C., Catapano A.L. (2016). 2016 European Guidelines on cardiovascular disease prevention in clinical practice: The Sixth Joint Task Force of the European Society of Cardiology and Other Societies on Cardiovascular Disease Prevention in Clinical Practice (constituted by representatives of 10 societies and by invited experts)Developed with the special contribution of the European Association for Cardiovascular Prevention & Rehabilitation (EACPR). Eur. Heart J..

[27] Smith S.C., Benjamin E.J., Bonow R.O., Braun L.T., Creager M.A., Franklin B.A. (2011). AHA/ACCF Secondary Prevention and Risk Reduction Therapy for Patients with Coronary and other Atherosclerotic Vascular Disease: 2011 update: a guideline from the American Heart Association and American College of Cardiology Foundation. Circulation.

[28] Montalescot G., Sechtem U., Achenbach S., Andreotti F., Arden C., Budaj A. (2013). 2013 ESC guidelines on the management of stable coronary artery disease: the Task Force on the management of stable coronary artery disease of the European Society of Cardiology. Eur. Heart J..

[29] Fihn S.D., Gardin J.M., Abrams J., Berra K., Blankenship J.C., Dallas A.P. (2012). 2012 ACCF/AHA/ACP/AATS/PCNA/SCAI/STS Guideline for the Diagnosis and Management of Patients With Stable Ischemic Heart Disease: Executive Summary: A Report of the American College of Cardiology Foundation/American Heart Association Task Force on Practice Guidelines, and the American College of Physicians, American Association for Thoracic Surgery, Preventive Cardiovascular Nurses Association, Society for Cardiovascular Angiography and Interventions, and Society of Thoracic Surgeons. J. Am. Coll. Cardiol..

[30] Long L, Anderson L, Dewhirst AM, He J, Bridges C, Gandhi M, et al. Exercise-based cardiac rehabilitation for adults with stable angina. Cochrane Database Syst. Rev. 2(2) (2018) Cd012786. 10.1002/14651858.CD012786.pub2 PMC6491173 29394453

[31] Archbold R.A. (2016). Comparison between National Institute for Health and Care Excellence (NICE) and European Society of Cardiology (ESC) guidelines for the diagnosis and management of stable angina: implications for clinical practice. Open Heart..

[32] Soga Y., Yokoi H., Ando K., Shirai S., Sakai K., Kondo K. (2010). Safety of early exercise training after elective coronary stenting in patients with stable coronary artery disease. Eur. J. Cardiovasc. Prev. Rehabil..

[33] Balady G.J., Leitschuh M.L., Jacobs A.K., Merrell D., Weiner D.A., Ryan T.J. (1992). Safety and clinical use of exercise testing one to three days after percutaneous transluminal coronary angioplasty. Am. J. Cardiol..

[34] Hu X., Zhang L., Wang H., Hao Q., Dong B., Yang M. (2017). Malnutrition-sarcopenia syndrome predicts mortality in hospitalized older patients. Sci. Rep..

[35] Woo J., Leung J., Morley J.E. (2015). Defining sarcopenia in terms of incident adverse outcomes. J. Am. Med. Dir. Assoc..

[36] Oerkild B., Frederiksen M., Hansen J.F., Simonsen L., Skovgaard L.T., Prescott E. (2011). Home-based cardiac rehabilitation is as effective as centre-based cardiac rehabilitation among elderly with coronary heart disease: results from a randomised clinical trial. Age Ageing.

[37] Smith K.M., McKelvie R.S., Thorpe K.E., Arthur H.M. (2011). Six-year follow-up of a randomised controlled trial examining hospital versus home-based exercise training after coronary artery bypass graft surgery. Heart.

[38] Leenders M., Verdijk L.B., van der Hoeven L., Adam J.J., van Kranenburg J., Nilwik R. (2013). Patients with type 2 diabetes show a greater decline in muscle mass, muscle strength, and functional capacity with aging. J. Am. Med. Dir. Assoc..

[39] Anbalagan V.P., Venkataraman V., Pradeepa R., Deepa M., Anjana R.M., Mohan V. (2013). The prevalence of presarcopenia in Asian Indian individuals with and without type 2 diabetes. Diabetes Technol. Ther..

[40] Srikanthan P., Hevener A.L., Karlamangla A.S. (2010). Sarcopenia exacerbates obesity-associated insulin resistance and dysglycemia: findings from the National Health and Nutrition Examination Survey III. PLoS One..

[41] Lu C.W., Yang K.C., Chang H.H., Lee L.T., Chen C.Y., Huang K.C. (2013). Sarcopenic obesity is closely associated with metabolic syndrome. Obes. Res. Clin. Pract..

[42] Ishii S., Tanaka T., Akishita M., Ouchi Y., Tuji T., Iijima K. (2014). Metabolic syndrome, sarcopenia and role of sex and age: cross-sectional analysis of Kashiwa cohort study. PLoS One..

[43] Huh J.Y. (2018). The role of exercise-induced myokines in regulating metabolism. Arch. Pharm. Res..

[44] Iemura S., Kawao N., Okumoto K., Akagi M., Kaji H. (2020). Role of irisin in androgen-deficient muscle wasting and osteopenia in mice. J. Bone Miner.Metab..

[45] Barbalho S.M., Flato U.A.P., Tofano R.J., Goulart R.A., Guiguer E.L., Detregiachi C.R.P. (2020). Physical Exercise and Myokines: Relationships with Sarcopenia and Cardiovascular Complications. Int. J. Mol. Sci..

[46] Araki E., Goto A., Kondo T., Noda M., Noto H., Origasa H. (2020). Japanese Clinical Practice Guideline for Diabetes 2019. Diabetol. Int..

[47] Kawano R., Takahashi F., Hashimoto Y., Okamura T., Miki A., Kaji A. (2021). Short energy intake is associated with muscle mass loss in older patients with type 2 diabetes: A prospective study of the KAMOGAWA-DM cohort. Clin. Nutr..

