# Supplementary material for: Impact of simple equation for estimating appendicular skeletal muscle mass in patients with stable coronary artery disease undergoing percutaneous coronary intervention
Source: Int J Cardiol Heart Vasc. 2022 Dec 12;44:101163. doi: 10.1016/j.ijcha.2022.101163 (PMC9762183; doi:10.1016/j.ijcha.2022.101163)
Supplement: Supplementary data 1 [file mmc1.pptx]

## Slide 1
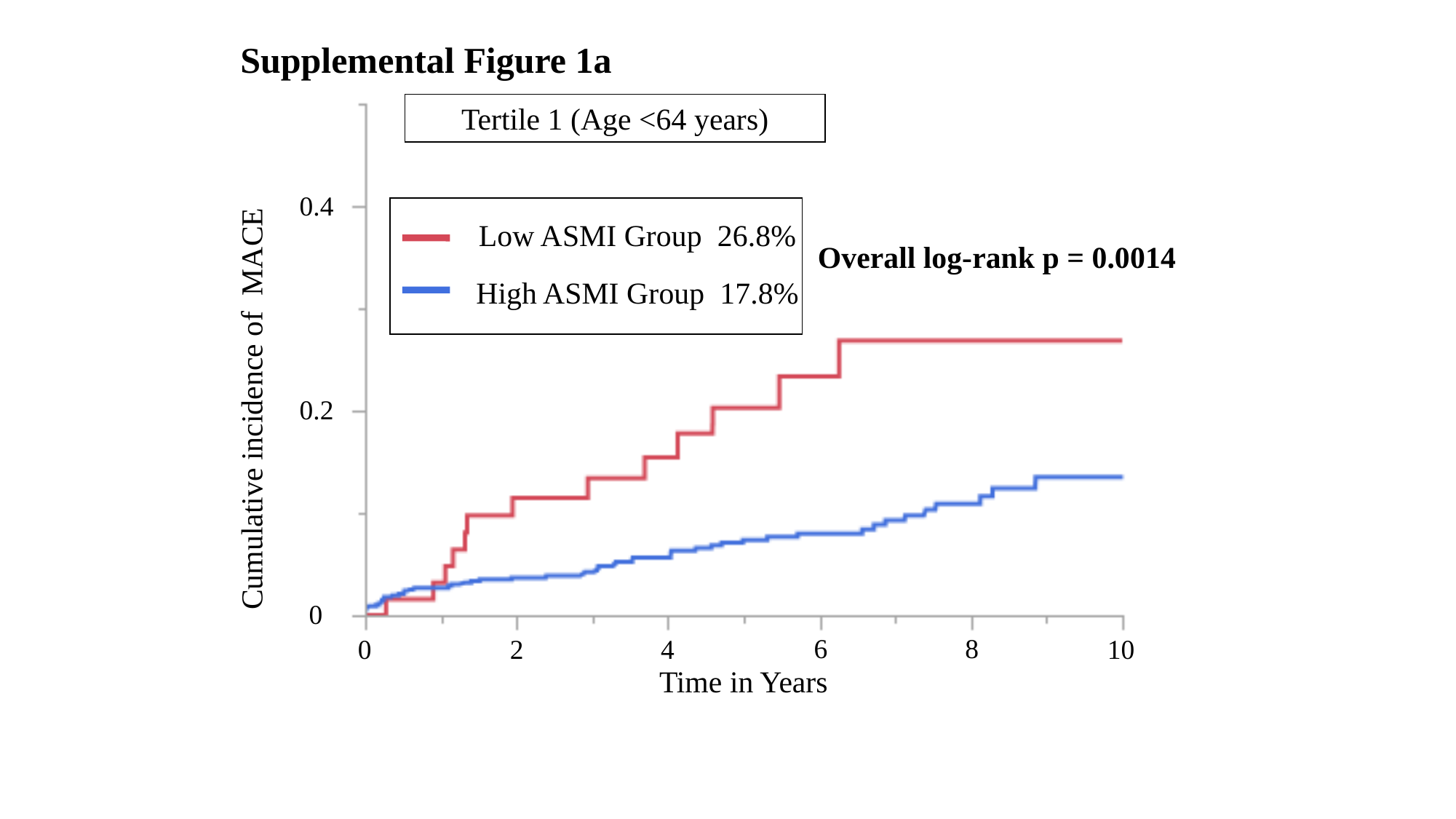

Supplemental Figure 1a
Tertile 1 (Age <64 years)
0.4
Overall log-rank p = 0.0014
0.2
Cumulative incidence of MACE
0
8
6
10
4
0
2
Time in Years
Low ASMI Group 26.8%
High ASMI Group 17.8%

## Slide 2
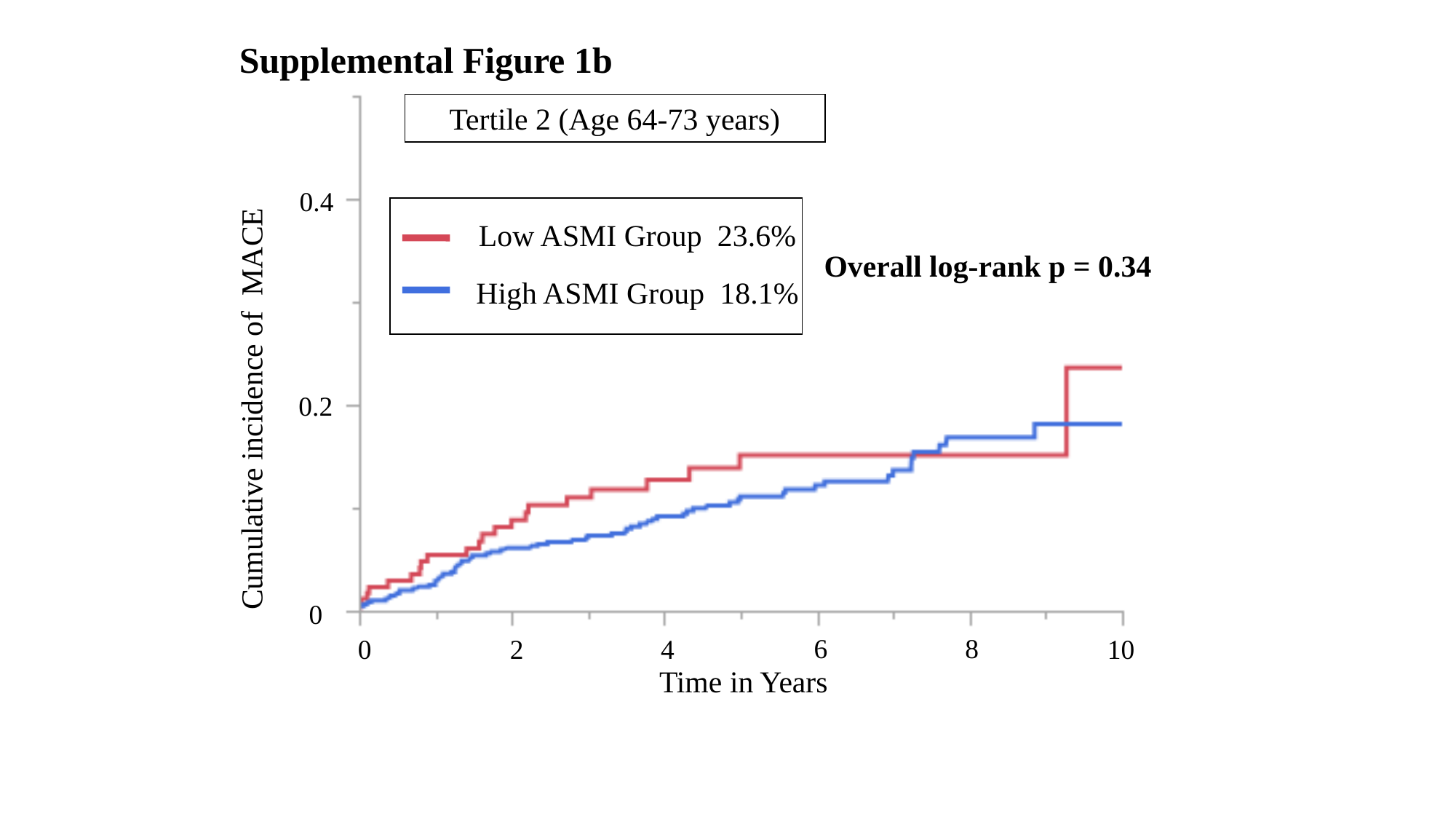

Supplemental Figure 1b
Tertile 2 (Age 64-73 years)
0.4
Overall log-rank p = 0.34
0.2
Cumulative incidence of MACE
0
8
6
10
4
0
2
Time in Years
Low ASMI Group 23.6%
High ASMI Group 18.1%

## Slide 3
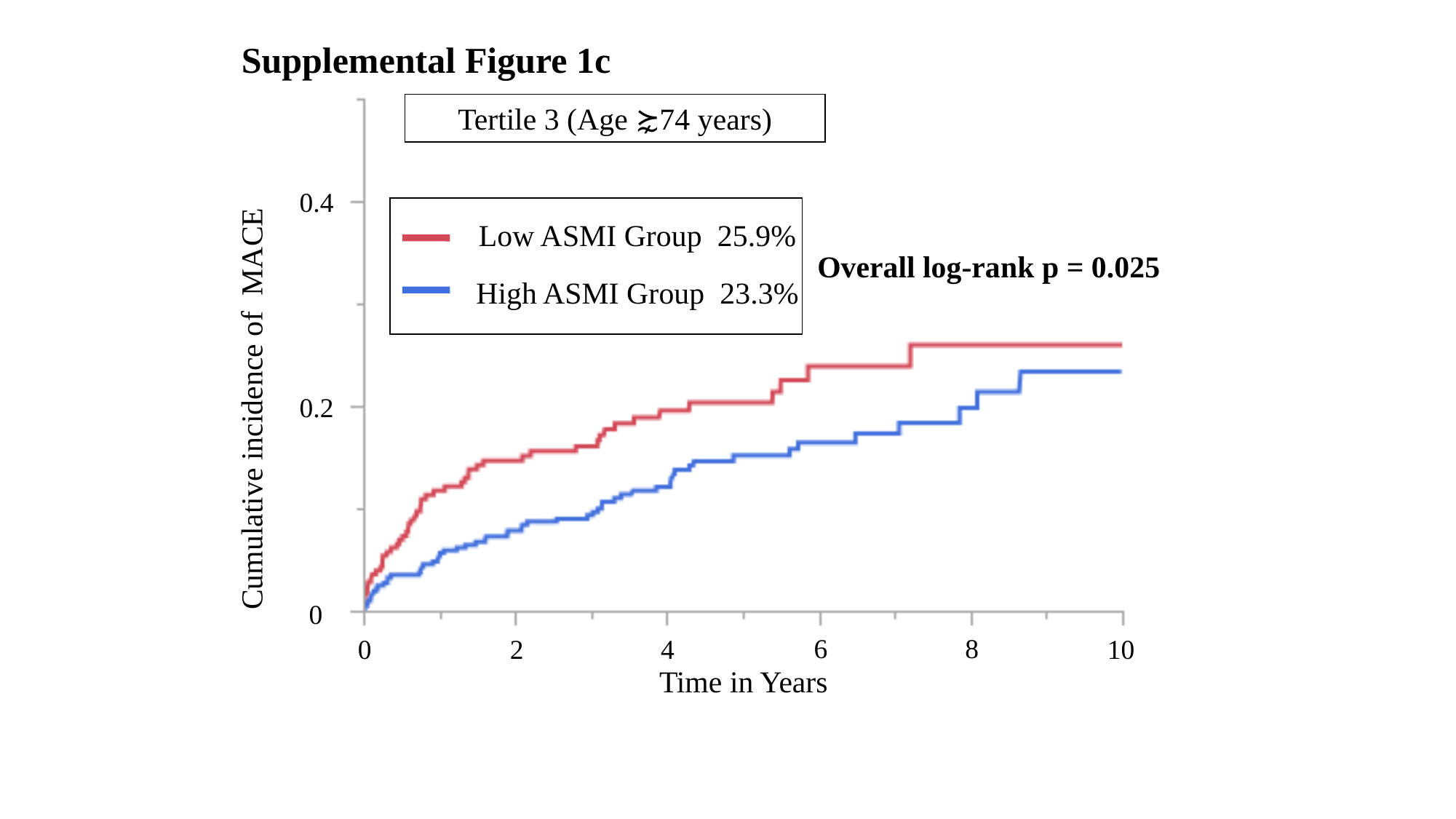

Supplemental Figure 1c
Tertile 3 (Age ⋩74 years)
0.4
Overall log-rank p = 0.025
0.2
Cumulative incidence of MACE
0
8
6
10
4
0
2
Time in Years
Low ASMI Group 25.9%
High ASMI Group 23.3%

## Slide 4
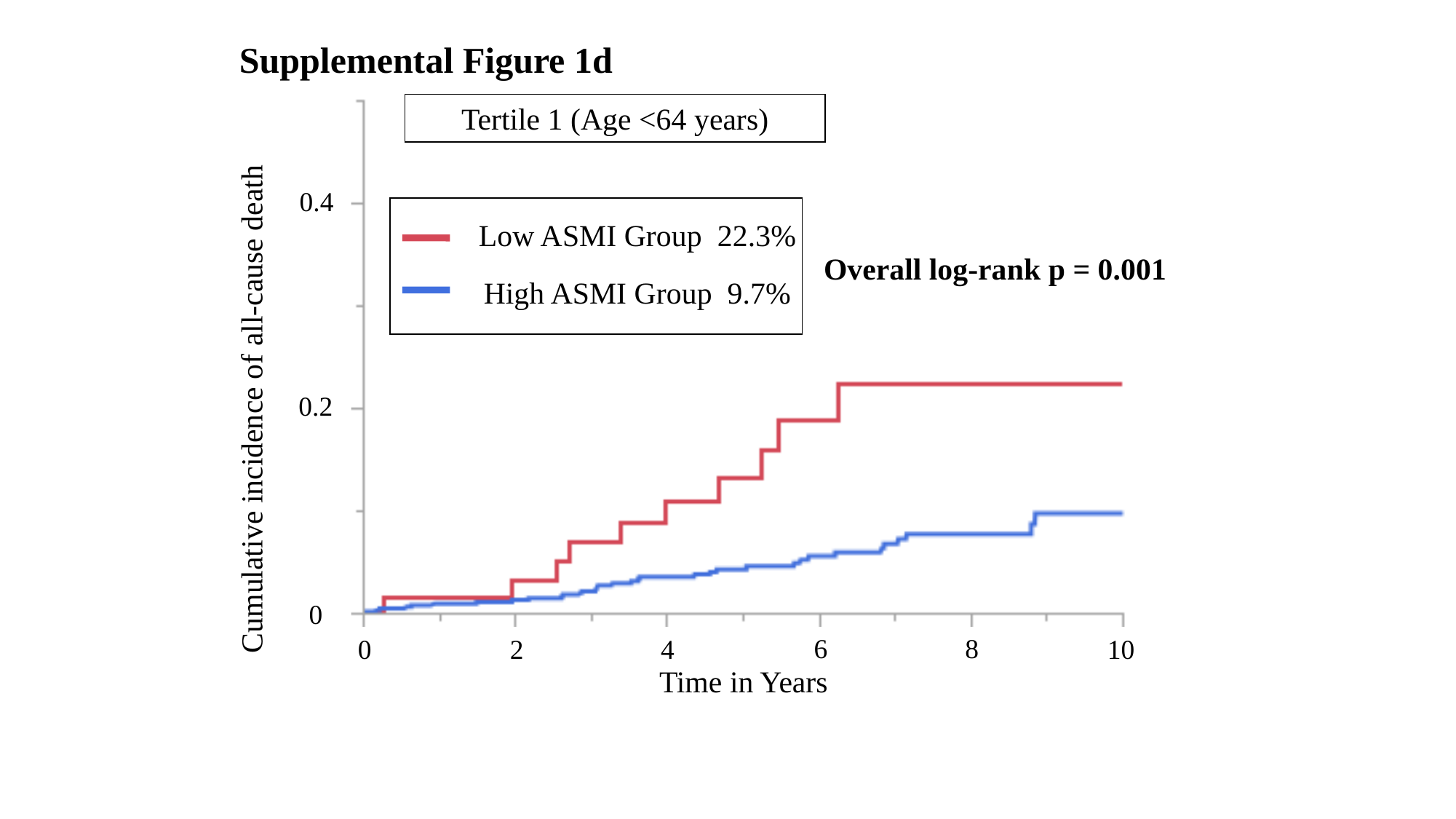

Supplemental Figure 1d
Tertile 1 (Age <64 years)
0.4
Overall log-rank p = 0.001
0.2
Cumulative incidence of all-cause death
0
8
6
10
4
0
2
Time in Years
Low ASMI Group 22.3%
High ASMI Group 9.7%

## Slide 5
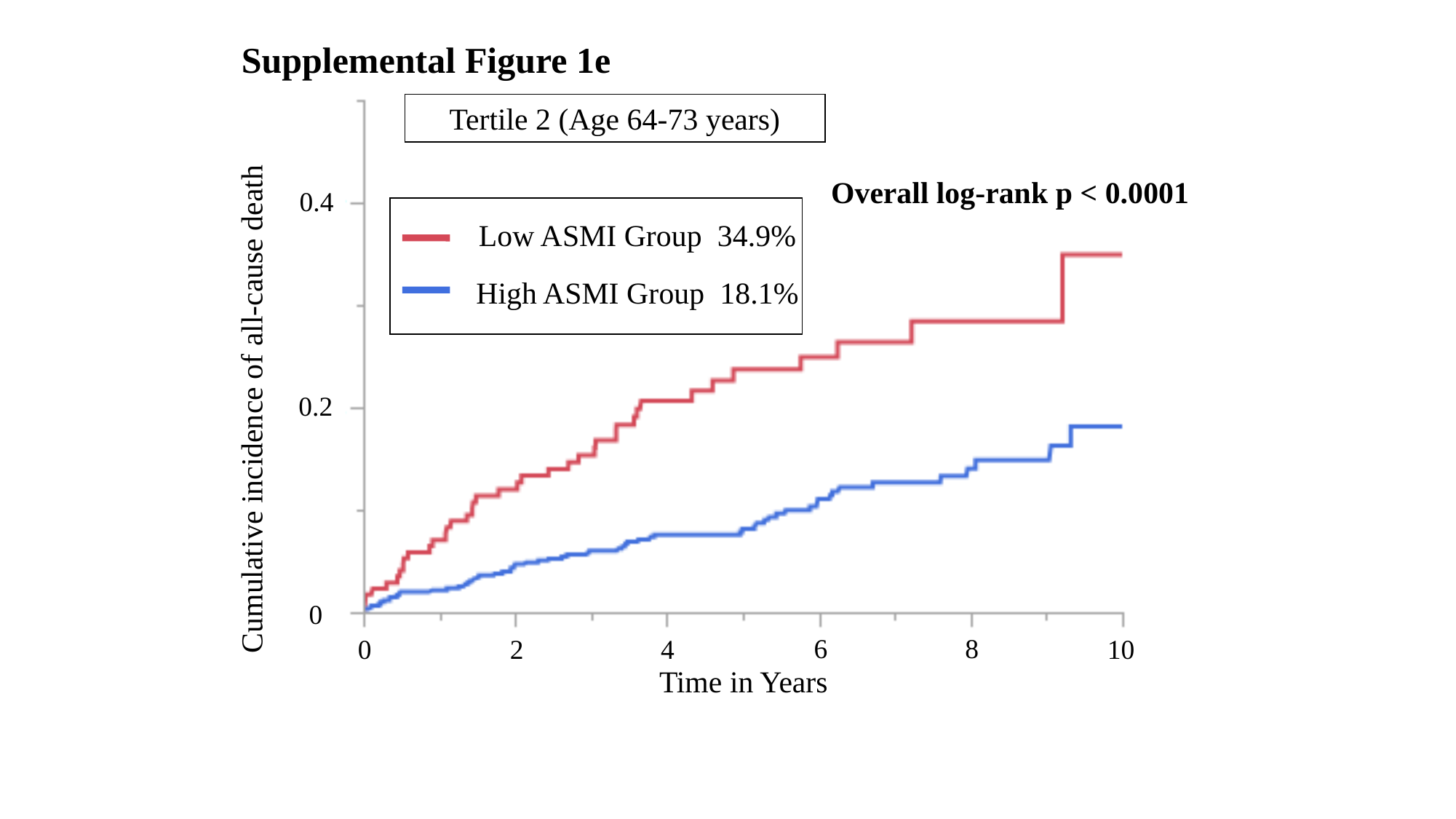

Supplemental Figure 1e
Tertile 2 (Age 64-73 years)
Overall log-rank p < 0.0001
0.4
0.2
Cumulative incidence of all-cause death
0
8
6
10
4
0
2
Time in Years
Low ASMI Group 34.9%
High ASMI Group 18.1%

## Slide 6
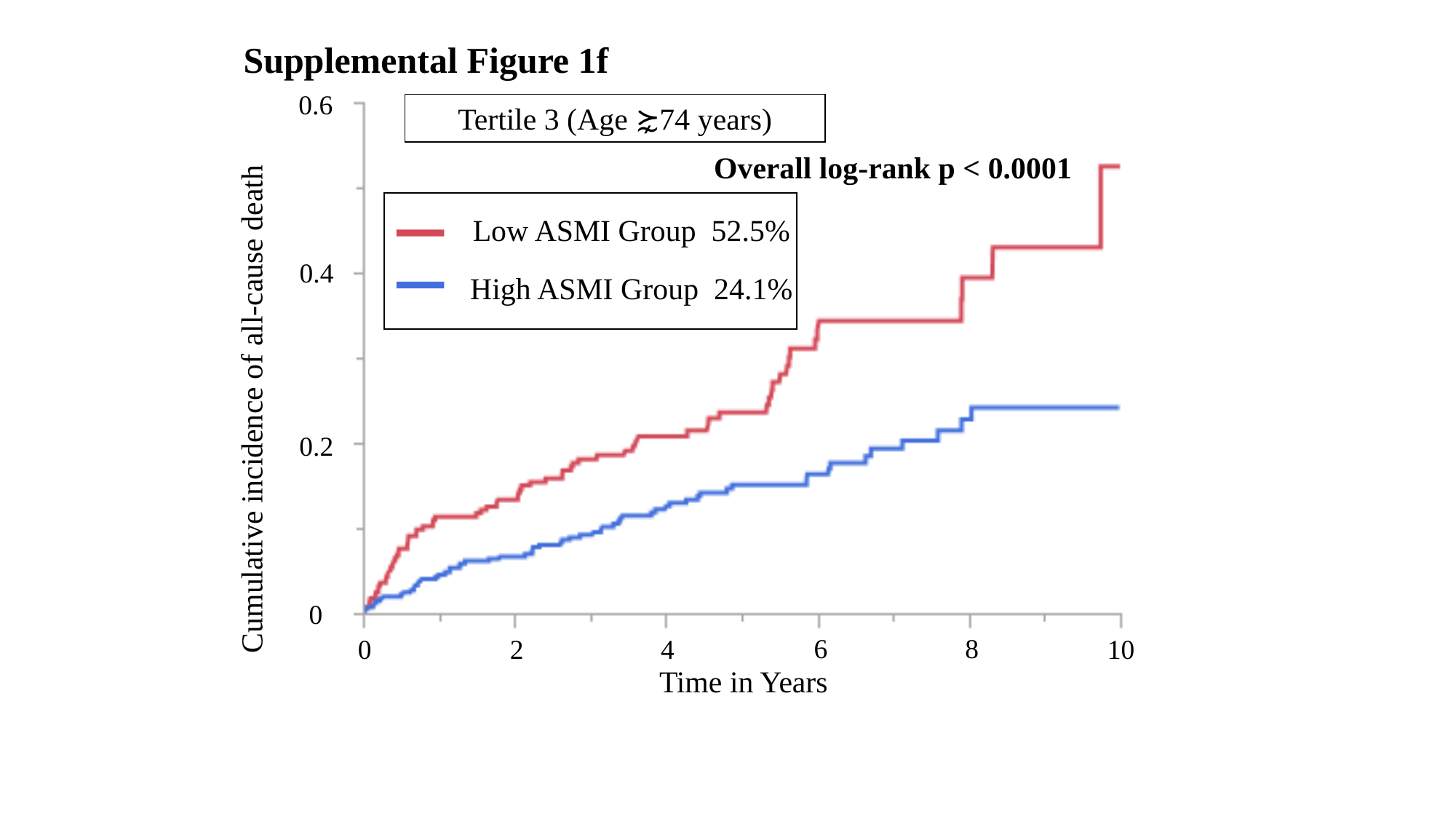

Supplemental Figure 1f
0.6
Tertile 3 (Age ⋩74 years)
Overall log-rank p < 0.0001
0.4
Cumulative incidence of all-cause death
0.2
0
8
6
10
4
0
2
Time in Years
Low ASMI Group 52.5%
High ASMI Group 24.1%
