# Supplementary material for: Impact of simple equation for estimating appendicular skeletal muscle mass in patients with stable coronary artery disease undergoing percutaneous coronary intervention
Source: Int J Cardiol Heart Vasc. 2022 Dec 12;44:101163. doi: 10.1016/j.ijcha.2022.101163 (PMC9762183; doi:10.1016/j.ijcha.2022.101163)
Supplement: Supplementary data 2 [file mmc2.docx]

Supplemental Table 1. Baseline clinical characteristics of patients stratified into age group

|  | **< 65 years** | |  | **≥65 years** | |  |
| --- | --- | --- | --- | --- | --- | --- |
|  | **Low ASMI** | **High ASMI** |  | **Low ASMI** | **High ASMI** |  |
|  | **(n = 73)** | **(n = 738)** | ***p*** | **(n = 450)** | **(n = 950)** | ***p*** |
| **Baseline characteristic** |  |  |  |  |  |  |
| ASMI, kg/m2 (Male) | 7.0 (7.0 – 7.1) | 8.3 (8.3 – 8.4) | <0.001 | 6.9 (6.9 – 7.0) | 7.9 (7.9 – 8.0) | <0.001 |
| ASMI, kg/m2 (Female) | 4.4 (2.7 – 6.1) | 6.2 (6.0 – 6.4) | 0.04 | 4.6 (4.6 – 4.7) | 5.8 (5.7 – 5.8) | <0.001 |
| Age, years | 59.1 ± 4.0 | 55.6 ± 7.1 | <0.001 | 76.4 ± 6.7 | 73.2 ± 5.8 | <0.001 |
| Male, n (%) | 72 (98.6) | 655 (88.8) | 0.001 | 364 (80.9) | 711 (74.8) | 0.01 |
| BMI, kg/m2 | 19.9 ± 1.4 | 26.1 ± 3.6 | <0.001 | 20.2 ± 1.8 | 25.1 ±2.6 | <0.001 |
| Hypertension, n (%) | 44 (60.2) | 491 (66.5) | 0.29 | 324 (72.0) | 752 (79.1) | 0.003 |
| Dyslipidemia, n (%) | 46 (63.1) | 613 (83.1) | <0.001 | 275 (61.1) | 728 (76.6) | <0.001 |
| Diabetes, n (%) | 27 (37.0) | 288 (39.0) | 0.73 | 191 (42.4) | 408 (43.0) | 0.86 |
| Current smoking, n (%) | 32 (43.8) | 267 (36.3) | 0.21 | 73 (16.3) | 137 (14.4) | 0.36 |
| CKD, n (%) | 16 (21.9) | 102 (13.8) | 0.08 | 179 (39.8) | 299 (31.5) | 0.002 |
| Family history of CAD, n (%) | 20 (27.4) | 265 (36.2) | 0.13 | 104 (23.2) | 231 (24.5) | 0.6 |
| ACS, n (%) | 28 (38.4) | 246 (33.3) | 0.39 | 118 (26.2) | 247 (26.0) | 0.93 |
| LVEF, % | 61.2 ± 12.8 | 60.8 ± 11.6 | 0.79 | 58.1 ± 13.8 | 61.8 ± 11.6 | <0.001 |
| Multivessel disease, n (%) | 42 (57.5) | 368 (50.6) | 0.25 | 295 (67.2) | 571 (60.9) | 0.02 |
| **Medication** |  |  |  |  |  |  |
| Aspirin, n (%) | 64 (87.8) | 686 (93.1) | 0.11 | 416 (92.7) | 892 (94.3) | 0.21 |
| β-blocker, n (%) | 35 (48.0) | 345 (47.6) | 0.95 | 176 (39.6) | 406 (43.2) | 0.21 |
| CCB, n (%) | 27 (37.0) | 228 (31.5) | 0.34 | 182 (41.0) | 438 (46.6) | 0.05 |
| ACE-I/ARB, n (%) | 26 (35.6) | 317 (43.7) | 0.18 | 203 (45.7) | 492 (52.3) | 0.02 |
| Statin, n (%) | 57 (78.1) | 626 (85.1) | 0.13 | 331 (73.9) | 778 (82.4) | <0.001 |
| **Baseline data** |  |  |  |  |  |  |
| HbA1c, % | 6.1 (5.8 – 6.3) | 6.3 (6.2 – 6.4) | 0.11 | 6.2 (6.1 – 6.3) | 6.3 (6,2 – 6.4) | 0.02 |
| TG, mg/dL | 111 (100 - 123) | 140 (154 - 174) | 0.002 | 97 (93 - 101) | 129 (122 - 135) | <0.001 |
| HDL-C, mg/dL | 48 (45 - 51) | 43 (42 - 44) | <0.001 | 48 (47 - 49) | 44 (43 - 45) | <0.001 |
| LDL-C, mg/dL | 91 (86 - 96) | 106 (103 - 109) | 0.002 | 98 (95 - 101) | 97 (95 - 99) | 0.63 |
| BNP, pg/mL | 183 (99 - 267) | 74 (59 - 90) | <0.001 | 253 (210 - 296) | 132 (112 - 154) | <0.001 |
| Alb, g/dL | 3.9 (3.5 – 4.4) | 4.0 (3.9 – 4.1) | 0.7 | 3.6 (3.5 – 3.7) | 3.8 (3.7 – 3.8) | 0.01 |
| eGFR, mL/min/1.73m2 | 64 (62 - 66) | 64 (62 - 66) | 0.03 | 61 (59 - 64) | 67 (65 - 68) | <0.001 |

ACE-I, angiotensin-converting enzyme inhibitors; ACS, acute coronary syndrome; Alb, albumin; ARB, angiotensin receptor blockers; ASMI, appendicular skeletal muscle index; BMI, body mass index; BNP, B-type natriuretic peptide; CAD, coronary artery disease; CKD, chronic kidney disease; eGFR, estimated glomerular filtration rate; HbA1c, hemoglobin A1c; HDL-C, high-density lipoprotein cholesterol; LDL-C, low-density lipoprotein cholesterol; LVEF, left ventricular ejection fraction; RIR, residual inflammatory risk; TG, triglycerides.
